# Evaluation of diagnostic efficacy of multimode ultrasound in BI-RADS 4 breast neoplasms and establishment of a predictive model

**DOI:** 10.3389/fonc.2022.1053280

**Published:** 2022-11-24

**Authors:** Yunhao Chen, Juerong Lu, Jie Li, Jingtang Liao, Xinyue Huang, Bo Zhang

**Affiliations:** Department of Ultrasonic Imaging, Xiangya Hospital, Central South University, Changsha, China

**Keywords:** breast neoplasms, ultrasound, elastography, contrast media, nomograms

## Abstract

**Objectives:**

To explore the diagnostic efficacy of ultrasound (US), two-dimensional and three-dimensional shear-wave elastography (2D-SWE and 3D-SWE), and contrast-enhanced ultrasound (CEUS) in breast neoplasms in category 4 based on the Breast Imaging Reporting and Data System (BI-RADS) from the American College of Radiology (ACR) and to develop a risk-prediction nomogram based on the optimal combination to provide a reference for the clinical management of BI-RADS 4 breast neoplasms.

**Methods:**

From September 2021 to April 2022, a total of 104 breast neoplasms categorized as BI-RADS 4 by US were included in this prospective study. There were 78 breast neoplasms randomly assigned to the training cohort; the area under the receiver-operating characteristic curve (AUC), 95% confidence interval (95% CI), sensitivity, specificity, positive predictive value (PPV), and negative predictive value (NPV) of 2D-SWE, 3D-SWE, CEUS, and their combination were analyzed and compared. The optimal combination was selected to develop a risk-prediction nomogram. The performance of the nomogram was assessed by a validation cohort of 26 neoplasms.

**Results:**

Of the 78 neoplasms in the training cohort, 16 were malignant and 62 were benign. Among the 26 neoplasms in the validation cohort, 6 were malignant and 20 were benign. The AUC values of 2D-SWE, 3D-SWE, and CEUS were not significantly different. After a comparison of the different combinations, 2D-SWE+CEUS showed the optimal performance. Least absolute shrinkage and selection operator (LASSO) regression was used to filter the variables in this combination, and the variables included Emax, Eratio, enhancement mode, perfusion defect, and area ratio. Then, a risk-prediction nomogram with BI-RADS was built. The performance of the nomogram was better than that of the radiologists in the training cohort (AUC: 0.974 vs. 0.863). In the validation cohort, there was no significant difference in diagnostic accuracy between the nomogram and the experienced radiologists (AUC: 0.946 vs. 0.842).

**Conclusions:**

US, 2D-SWE, 3D-SWE, CEUS, and their combination could improve the diagnostic efficiency of BI-RADS 4 breast neoplasms. The diagnostic efficacy of US+3D-SWE was not better than US+2D-SWE. US+2D-SWE+CEUS showed the optimal diagnostic performance. The nomogram based on US+2D-SWE+CEUS performs well.

## Introduction

According to the data released by the International Agency for Research on Cancer in 2020, female breast cancer has replaced lung cancer as the most commonly diagnosed cancer globally and has the highest mortality rate. It poses a serious threat to women’s health and lives and brings about tremendous challenges to public health worldwide (1). Ultrasound (US) is the most important method for breast cancer screening in China. According to the fifth edition of Breast Imaging Reporting and Data System (BI-RADS) from the American College of Radiology (ACR), category 4 breast neoplasms are further classified into 4A, 4B, and 4C subcategories, with a large risk span (2%–95%), and biopsy or surgery is usually recommended. However, Ultrasound images of benign and malignant tneoplasms frequently overlap, resulting in many benign tumors that can be followed up to be biopsied as malignant neoplasms. (2), with low specificity. As a supplement to conventional ultrasound, shear-wave elastography (SWE) and contrast-enhanced ultrasound (CEUS) can provide more information for clinicians to tailor treatment plans (3, 4). Numerous clinical studies identified the potential value of SWE and CEUS in differentiating benign and malignant breast neoplasms, which might reduce the biopsy rate of breast neoplasms (4–6). However, to our knowledge, it is very rare to study the diagnostic efficacy of two-dimensional shear-wave elastography (2D-SWE), three-dimensional shear-wave elastography (3D-SWE), CEUS, and their combination in BI-RADS 4 neoplasms. Based on this situation, we conducted this study and developed a well-performed risk-prediction nomogram, hoping to provide a reference for the clinical management of BI-RADS 4 breast neoplasms.

## Materials and methods

### Patients

**Figure 1:**
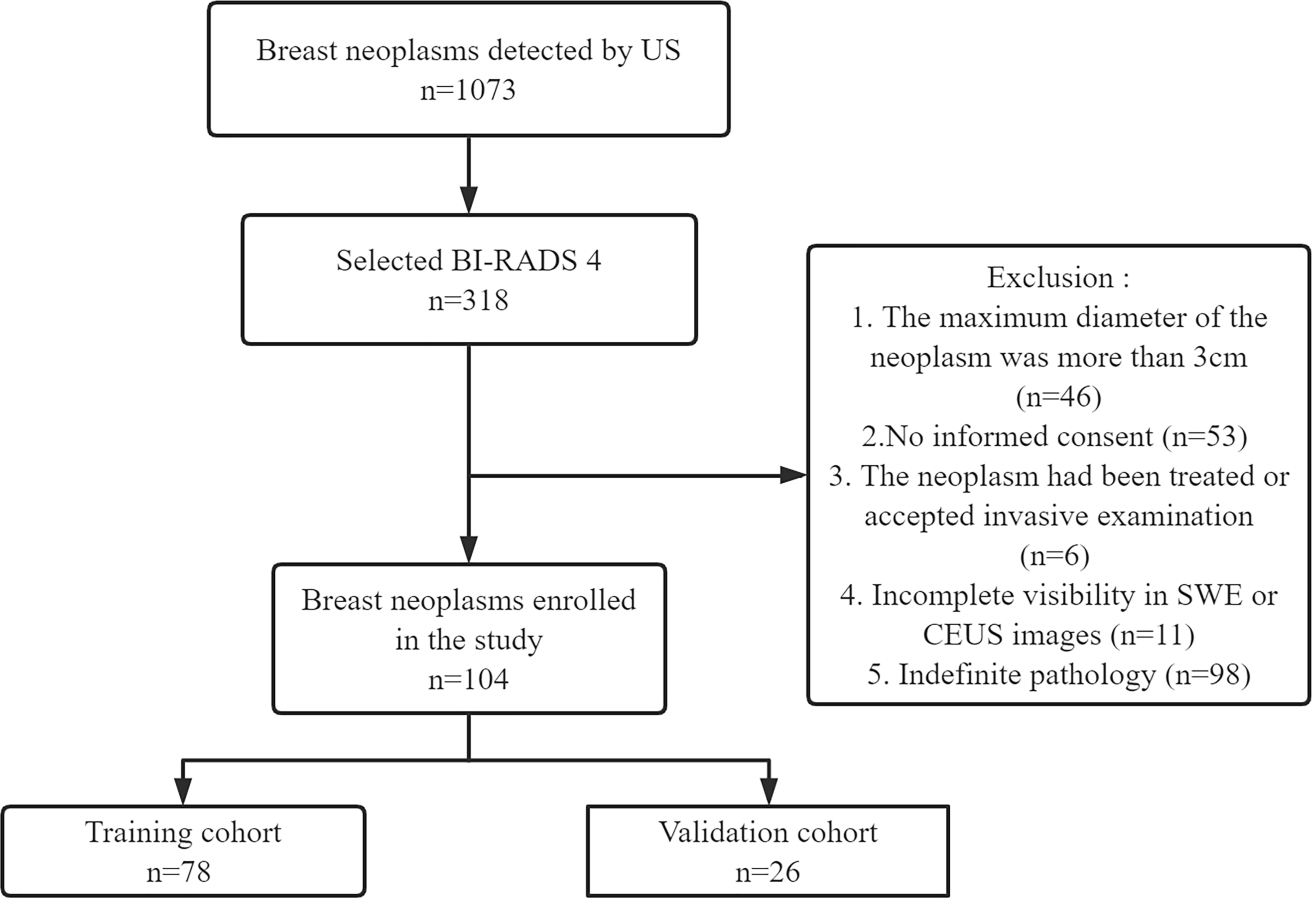
The flowchart of this study.

This prospective study (clinical trial ChiCTR2100050604) was approved by our hospital (No. 202010143). The study was conducted in accordance with the Declaration of Helsinki. Informed consent was obtained from all patients at enrollment. From September 2021 to April 2022, 101 female patients with BI-RADS 4 breast neoplasms (104 neoplasms) were recruited. According to the ratio of 4:1, 78 out of 104 neoplasms were randomly selected as the training cohort and the remaining 26 neoplasms were chosen as the validation cohort. The training cohort was comprised of 78 neoplasms in 75 women. Among them, the bilateral breast of three patients had BI-RADS 4 neoplasms. The validation cohort included 26 neoplasms in 26 women. Pathology was confirmed by surgery or vacuum-assisted biopsy. The exclusion criteria were as follows: 1) the maximum diameter of the neoplasm was more than 3 cm (limited by the SWE region of interest box), 2) no informed consent was provided, 3) the neoplasm had been treated or accepted invasive examination, 4) there was incomplete visibility in SWE or CEUS images, and 5) there was indefinite pathology. The study flowchart is shown in **Figure 1**.

### US data acquisition and analysis

US examinations were performed with the Aixplorer ultrasound system (SuperSonic Imagine, Aix-en-Provence, France) equipped with a 5–14-MHz linear array transducer. All of the neoplasms were examined and assessed by a radiologist with over 20 years of experience in breast US examination. The patients were lying in the supine position. Imaging parameters were adjusted to optimally visualize the target neoplasm with the largest diameter. US images were obtained on the plane and vertical section of the target neoplasm. Additional images containing important features (margin, calcification, blood, etc.) were also stored. After careful observation of the B-mode image of the neoplasm, the basic characteristics of the neoplasm (size, margin, shape, internal echo, calcification, blood, aspect ratio) were recorded. Each neoplasm was described as complying with the fifth edition of the ACR BI-RADS US atlas and was ultimately assigned a category (BI-RADS 4A, 4B, or 4C).

### SWE data acquisition and analysis

All SWE examinations were performed with the Aixplorer ultrasound system (SuperSonic Imagine, Aix-en-Provence, France) equipped with a 5–14-MHz linear array transducer (for 2D-SWE examination) and a 5–16-MHz dedicated mechanical volumetric transducer (for 3D-SWE examination). The examination was carried out by a radiologist with at least 3-year experience in breast SWE. The region of interest (ROI) box of the SWE color map covered the neoplasm and sufficient surrounding glands. 2D-SWE and 3D-SWE images were obtained on the plane and vertical section of the target neoplasm with the largest diameter (penetration mode was selected in case of poor condition), and the default stiffness ranged from 0 to 180 kPa. Patients were required to hold their breath after the slightest pressure, and the probe was applied with a generous layer of US gel to reduce artificial stiffness. In order to avoid the influence of uncertain factors such as contrast agent perfusion, SWE was completed before CEUS.

Among the stored 2D-SWE images, three high-quality images were selected for analysis in transverse and sagittal planes of the largest diameter of the target neoplasm. The color distribution of the neoplasms was observed for qualitative analysis. The big round ROI (covering the whole neoplasm) was used for the analysis of the neoplasms as well as the recording of the maximum stiffness (Emax), mean stiffness (Emean), and standard deviation (Esd). Two small round ROIs of 2 mm diameter were used to measure the neoplasm-to-fat/gland elasticity ratio (Eratio). They were placed on the hardest part of the neoplasm and the surrounding glands or fat with the same depth, respectively, and recorded as eEmax, eEmean, eEsd, and eEratio. Among the stored 3D-SWE images, three high-quality images were selected for analysis in the transverse, sagittal, and coronal planes, respectively. The stiffest part was searched in the multislice and multiplanar views of the neoplasm. The same measuring method as 2D-SWE was employed for 3D-SWE. All values were averages of three repeated measurements and recorded as tEmax, tEmean, tEsd, and tEratio.

### CEUS data acquisition and analysis

All CEUS examinations were performed with Resona 7S or R9 devices (Mindray Medical, Shenzhen, China) equipped with a 9–3-MHz linear-array transducer. CEUS was performed by a radiologist who had more than 15 years of CEUS examination experience. Real-time CEUS was performed on the largest section of the neoplasm and sufficient surrounding glands. A total of 4.8 ml of SonoVue (Bracco, Italy) was quickly injected through the cubital vein, and 5 ml of saline was used for flushing the syringe before and after injection. During the examination, the patients were required to maintain smooth breathing and posture. The dynamic images were recorded for 90 s.

Enhancement mode, enhancement boundary, artery enhancement intensity, perfusion defect, perforator vessels, and venous enhancement intensity of the neoplasms were recorded for qualitative analysis. The time–intensity curve (TIC) was plotted to quantitatively analyze the part of the neoplasm with higher enhancement. Base intensity (BI), peak intensity (PI), time to peak (TTP), peak intensity halving time (DT/2), and the area under the TIC curve (tAUC) were recorded. Playing back the dynamic image, when the enhancement intensity of the neoplasm reached the peak, the boundaries of the B-mode image and contrast image were tracked, respectively, and the area ratios before and after contrast were calculated. When the neoplasm showed iso-enhancement with the surrounding glands and the boundary was unclear, the default area ratio was 1. All values were averages of three repeated measurements.

All image analyses were reviewed by at least two radiologists, and any differences were resolved through consultation.

### Development and validation of the nomogram

The diagnostic efficacy of US, 2D-SWE, 3D-SWE, CEUS alone, and their combination for BI-RADS 4 breast neoplasms was compared and analyzed, and the best combination was selected. The least absolute shrinkage and selection operator (LASSO) regression was employed to minimize the multicollinearity of the ultrasonic features in the best combination. Five ultrasonic features with the highest absolute value of coefficient were screened and combined with the BI-RADS classification to develop a risk-prediction nomogram. The calibration curve, decision curve analysis (DCA), and receiver-operating characteristic (ROC) curve were employed to validate the performance of the nomogram.

### Statistical analysis

SPSS Statistics (version 25.0), R software (version 3.6.3), and MedCalc (version 19.5.6) were used for data analysis. Continuous variables were expressed as the mean ± SD and analyzed by *t*-test or rank-sum test. Categorical variables were presented as frequencies and percentages and evaluated with the *χ*² test or Fisher’s exact test. The optimal cutoff value was obtained in the ROC curve completed by SPSS 25. The reported statistical significance levels were two-sided, and *P <*0.05 was considered statistically significant. LASSO regression was used to select significant features. The R software was employed to develop and assess the nomogram; the calibration curve and DCA were used for the evaluation of the nomogram. The area under the receiver-operating characteristic curve (AUC) was compared by MedCalc 19.5.6.

## Results

### Patient characteristics

Of the 78 breast neoplasms in the training cohort, 16 were malignant (mean age ± standard deviation, 44.63 ± 9.16 years) and 62 were benign (42.77 ± 9.54 years), classified into BI-RADS 4A (56 cases), BI-RADS 4B (18 cases), and BI-RADS 4C (4 cases). Among the 26 breast neoplasms in the validation cohort, 6 were malignant (46.67 ± 6.41 years) and 20 were benign (44.00 ± 11.53 years), involving BI-RADS 4A (22 cases), BI-RADS 4B (2 cases), and BI-RADS 4C (2 cases). The pathology results are shown in **Table 1**.

**Table 1:** Pathological diagnosis of 104 category BI-RADS 4 breast neoplasms.

Pathology	Training cohort (*n* = 78)		Validation cohort (*n* = 26)	
	Malignant	Benign	Malignant	Benign
Total	*n* = 16	*n* = 62	*n* = 6	*n* = 20
Fibroadenoma		*n* = 31		*n* = 13
Adenosis		*n* = 21		*n* = 5
Intraductal papilloma		*n* = 6		*n* = 1
Abscess or mastitis		*n* = 2		*n* = 1
Other benign diagnoses		*n* = 2		
Ductal carcinoma *in situ*	*n* = 1		*n* = 1	
Invasive ductal cancer	*n* = 4			
Lobular carcinoma			*n* = 1	
Medullary carcinoma	*n* = 2			
Mucinous carcinoma	*n* = 1			
Other invasive cancer	*n* = 8		*n* = 4	

### US characteristics

In the training cohort, statistical differences could be found in the calcification and in the blood in the benign and malignant neoplasms, and the calcification and margin were significantly different in the validation cohort.

### Diagnostic efficacy of 2D-SWE and 3D-SWE

Statistical differences could be found in the qualitative (2D-SWE/3D-SWE color distribution) and quantitative features (e/tEmax, e/tEmean, e/tEsd, and e/tEratio) in the training cohort (**Table 2**). The cutoff value (Youden index) was obtained from the ROC curve. With eEmax ≥57.38 kPa, eEmean ≥25.83 kPa, eEsd ≥9.18 kPa, and eEratio ≥6.21, polychrome/hard ring signs were recorded as malignant neoplasms in 2D-SWE. With tEmax ≥68.56 kPa, tEmean ≥26.01 kPa, tEsd ≥10.69 kPa, and tEratio ≥6.89, polychrome/hard ring signs were classified as malignant neoplasms in 3D-SWE. When one of the qualitative or quantitative criteria was positive, the neoplasm was assessed as malignant.

**Table 2:** Ultrasonographic features of category BI-RADS 4 breast neoplasms in the training cohort.

	Malignant (*n* = 16)	Benign (*n* = 62)	*P*-value
Size (mm)	21.44 ± 5.92	11.43 ± 5.15	<0.001*
Margin			0.094
Smooth	4 (25.00%)	31 (50.00%)	
Angular/irregular	12 (75.00%)	31 (50.00%)	
Shape			0.169
Regular	1 (6.20%)	15 (24.20%)	
Irregular	15 (93.80%)	47 (75.80%)	
Internal echo			1.000
Hypoecho	15 (93.80%)	58 (93.50%)	
Mixed-echo	1 (6.20%)	4 (6.50%)	
Calcification			0.011*
None	7 (43.80%)	49 (79.00%)	
Yes	9 (56.20%)	13 (21.00%)	
Aspect ratio			0.080
>1	12 (75.00%)	57 (91.90%)	
<1	4 (25.00%)	5 (8.10%)	
Blood			<0.001*
Absent	0 (0.00%)	34 (54.80%)	
Presence	16 (100.00%)	28 (45.20%)	
2D-SWE color distribution			<0.001*
Blue/inhomogeneous blue	1 (6.2%)	57 (91.9%)	
Polychrome/hard ring sign	15 (93.8%)	5 (8.1%)	
eEmean (kPa)	50.35 ± 20.86	14.04 ± 1.01	<0.001*
eEmax (kPa)	201.40 ± 77.41	33.71 ± 3.48	<0.001*
eEsd (kPa)	39.93 ± 17.68	5.81 ± 0.69	<0.001*
eEratio	13.38 ± 6.53	2.57 ± 0.29	<0.001*
3D-SWE color distribution			<0.001*
Blue/inhomogeneous blue	1 (6.2%)	55 (88.7%)	
Polychrome/hard ring sign	15 (93.8%)	7 (11.3%)	
tEmean (kPa)	40.12 ± 15.24	13.04 ± 0.77	<0.001*
tEmax (kPa)	181.86 ± 42.00	41.56 ± 3.44	<0.001*
tEsd (kPa)	33.57 ± 9.97	7.01 ± 0.61	<0.001*
tEratio	14.74 ± 4.93	3.04 ± 0.26	<0.001*
Enhancement mode			<0.001*
Homogeneous	2 (12.5%)	27 (43.5%)	
Heterogeneous	14 (87.5%)	35 (56.5%)	
Enhancement boundary			0.004*
Clear	14 (87.5%)	28 (45.2%)	
Not clear	2 (12.5%)	34 (54.8%)	
Artery enhancement intensity			0.011*
Low	1 (6.2%)	22 (35.5%)	
Middle	0 (0.0%)	4 (6.5%)	
High	15 (93.8%)	36 (58.1%)	
Perfusion defect			<0.001*
Absent	10 (62.5%)	60 (96.8%)	
Present	6 (37.5%)	2 (3.2%)	
Perforator vessels			<0.001*
Absent	3 (18.8%)	55 (88.7%)	
Present	13 (81.2%)	7 (11.3%)	
Venous enhancement intensity			0.758
Low	9 (56.3%)	27 (43.6%)	
Middle	2 (12.5%)	19 (30.6%)	
High	5 (31.2%)	16 (25.8%)	
BI (dB)	6.40 ± 3.89	5.89 ± 3.49	0.447
TTP (s)	17.55 ± 8.07	>14.69 ± 4.70	
PI (dB)	21.55 ± 4.11	17.06 ± 6.74	0.016*
DT/2 (s)	57.21 ± 9.18	53.99 ± 13.44	0.124
tAUC	1,326.83 ± 290.90	1,016.60 ± 468.72	0.013*
Area ratio	1.30 ± 0.18	1.06 ± 0.12	0.005*

*P-value indicates significant difference.

### Diagnostic efficacy of CEUS

Significant differences existed in arterial enhancement intensity, enhancement mode, enhancement boundary, perfusion defect, and perforator vessel in the qualitative features of CEUS in the training cohort (*P* < 0.05). Malignant neoplasms were characterized by high-intensity enhancement in the arterial phase, heterogeneous enhancement mode, unclear enhancement boundary, perfusion defect, and perforating vessels. The quantitative features of PI, tAUC, and area ratio were significantly higher in malignant neoplasms than in benign ones, with statistical differences (**Table 2**). The cutoff value was obtained by the ROC curve. Neoplasms with PI ≥16.57 dB, tAUC ≥866.23, and area ratio ≥1.20 were considered malignant. When one of the qualitative or quantitative criteria was positive, neoplasms were classified as malignant.

### Diagnostic efficacy of the different multimode ultrasound combinations in BI-RADS 4 neoplasms

**Figure 2:**
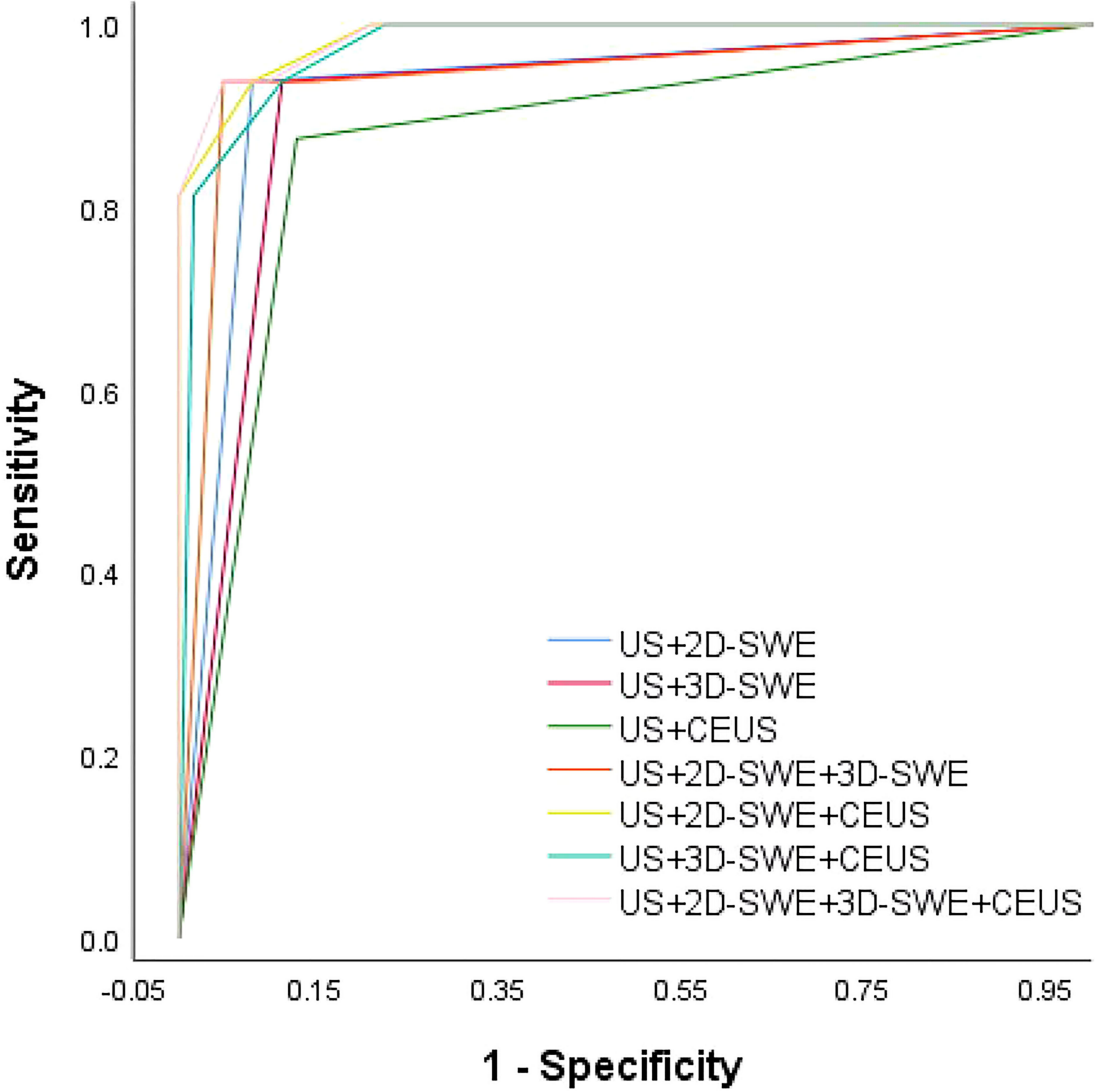
ROC curve of the multimode ultrasound combination.

No significant difference was observed between the qualitative and quantitative features of US, 2D-SWE, 3D-SWE, and CEUS in BI-RADS 4 neoplasms in the training cohort (**Table 3**). The ROC curves of the multimode ultrasound combinations are shown in **Figure 2**. Differences in the diagnostic efficacy of the multimode ultrasound combination are shown in **Table 4**, and there was no significant difference in AUC values among US+2D-SWE, US+3D-SWE, and US+CEUS. US+2D-SWE+CEUS could significantly improve the diagnostic efficacy of US+2D-SWE, US+3D-SWE, and US+CEUS (*P* < 0.05), and there was no significant difference compared with US+2D-SWE+3D-SWE, US+3D-SWE+CEUS, and US+2D-SWE+3D-SWE+CEUS. US+2D-SWE+CEUS showed the best performance (AUC 0.986, sensitivity 93.75, and specificity 91.94).

**Table 3:** The diagnostic performance of the quantitative and qualitative analyses of 2D-SWE, 3D-SWE, and CEUS in the training cohort.

	AUC	95% CI	Sensitivity	Specificity	PPV	NPV	*P*-value
CEUS qualitative	0.794	0.688, 0.878	75	83.87	54.5	92.9	*P* = 0.635
CEUS quantitative	0.835	0.733, 0.909	75	91.94	70.6	93.4	
2D-SWE qualitative	0.928	0.847, 0.974	93.75	91.94	75	98.3	*P* = 0.977
2D-SWE quantitative	0.929	0.848, 0.975	87.5	98.39	93.3	96.8	
3D-SWE qualitative	0.912	0.826, 0.964	93.75	88.71	68.2	98.2	*P* = 0.639
3D-SWE quantitative	0.929	0.848, 0.975	87.5	98.39	93.3	96.8	

**Table 4:** Differences in the diagnostic efficacy of the multimode ultrasound combination in the training cohort.

	①+②	①+③	①+④	①+②+③	①+②+④	①+③+④	①+②+③+④
①+②	/	0.415	0.375	0.238	0.029*	0.109	0.024*
①+③	0.415	/	0.530	0.053	0.012*	0.018*	0.008*
①+④	0.375	0.530	/	0.530	0.015*	0.024*	0.016*
①+②+③	0.238	0.053	0.530	/	0.112	0.249	0.091
①+②+④	0.029*	0.012*	0.015*	0.112	/	0.279	0.479
①+③+④	0.109	0.018*	0.024*	0.249	0.279	/	0.194
①+②+③+④	0.024*	0.008*	0.016*	0.091	0.479	0.194	/

*P-value indicates significant difference. ①: US; ②: 2D-SWE; ③: 3D-SWE; ④: CEUS.

### Diagnostic efficacy of the radiologist and the nomogram

**Figure 3:**
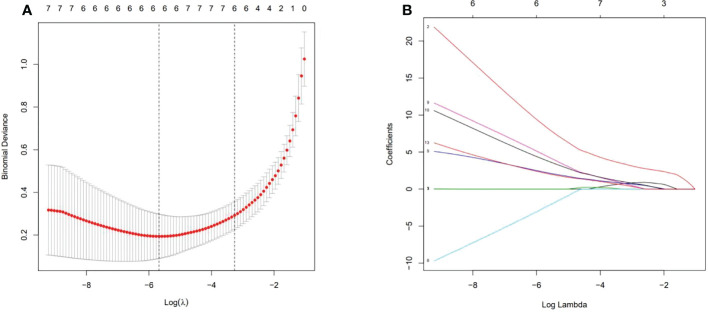
Ultrasonographic feature selection using the least absolute shrinkage and selection operator (LASSO) regression in the training cohort. **(A)** Lambda (*λ*) selection in the LASSO model used 10-fold cross-validation *via* minimum criteria. The value of *λ* was used to select features. Vertical lines were drawn at the optimal values using the minimum criteria and the 1-SE criteria. The optimal value of 0.0034 was selected. **(B)** Coefficient profiles of the 13 features.

**Figure 4:**
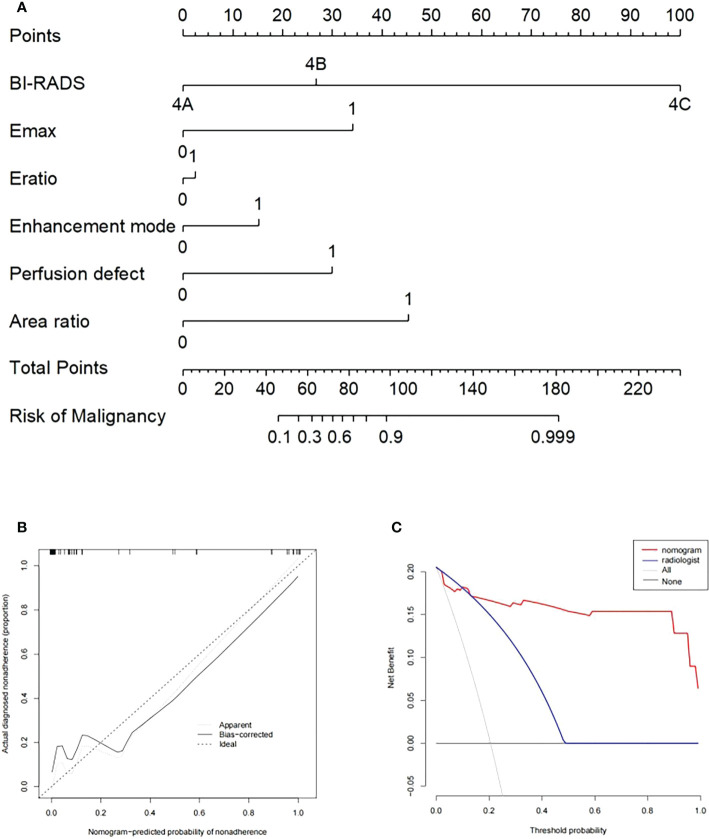
**(A)** Nomogram with selected ultrasonographic feature and the BI-RADS category incorporated. Performance and clinical usefulness evaluation of the nomogram. **(B)** Calibration curves for the nomogram in the training cohort. **(C)** Decision curve analysis (DCA) derived from the training cohort.

**Figure 5:**
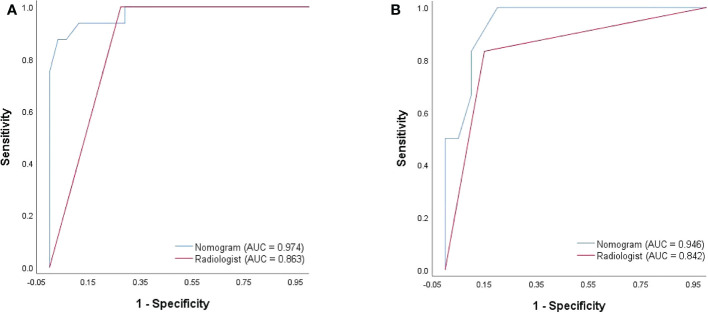
ROC curves of the nomogram and the radiologist’s diagnosis derived from the training cohort **(A)** and the validation cohort **(B)**.

After 2D-SWE, 3D-SWE, and CEUS examination, the radiologist reclassified the neoplasms as benign type in BI-RADS 3 (45/78, 18/26) and malignant type in BI-RADS 4a and above (33/78, 8/26). LASSO regression was used to filter the variables in this combination (**Figure 3**), and the variables included eEmax, eEratio, enhancement mode, perfusion defect, and area ratio. Then, a risk-prediction nomogram with BI-RADS was built (**Figure 4A**). The calibration curve was drawn to explore the prediction accuracy of the nomogram (**Figure 4B**). DCA was used to evaluate the clinical practicability of the nomogram, identifying its high prediction accuracy and clinical value (**Figure 4C**). The ROC curve was employed to evaluate the ability of the nomogram and the radiologists to diagnose benign and malignant neoplasms (**Figure 5A**). The performance of the nomogram was better than that of the radiologists, with statistical significance of AUC differences (*P* < 0.001), especially the significantly higher specificity (98.39 vs. 72.58) and PPV (92.9 vs. 48.5) (**Table 5**).

To better evaluate the diagnostic efficacy of this nomogram for clinical application, only the ultrasonographic features in the nomogram were included in the validation cohort (**Table 6**). The ROC curve of the validation cohort was drawn and compared with that of the radiologist (**Figure 5B**). The nomogram had a similar performance but a higher PPV compared with the radiologists (71.4 vs. 62.5) (**Table 5**).

**Table 5:** The diagnostic performance of the US+2D-SWE+CEUS, nomogram, and radiologist.

		AUC	95% CI	Sensitivity	Specificity	PPV	NPV
US+2D-SWE+CEUS		0.986	0.964, 1.000	93.75	91.94	75	98.3
Training cohort	Nomogram	0.975	0.911, 0.997	81.25	98.39	92.9	95.3
	Radiologist	0.863	0.766, 0.930	100	72.58	48.5	100
Validation cohort	Nomogram	0.946	0.780, 0.997	83.33	90	71.4	94.7
	Radiologist	0.842	0.646, 0.954	83.33	85	62.5	94.4

**Table 6:** Ultrasonographic features of category BI-RADS 4 breast neoplasms in the validation cohort.

	Malignant (*n* = 6)	Benign (*n* = 20)	*P*-value
US			
Size (mm)	17.83 ± 3.92	14.09 ± 6.28	0.183
Margin			0.040*
Smooth	1 (16.70%)	17 (85.00%)	
Angular/irregular	5 (83.30%)	3 (15.00%)	
Shape			0.298
Regular	0 (0.00%)	5 (25.00%)	
Irregular	6 (100.00%)	15 (75.00%)	
Internal echo			1.000
Hypoecho	6 (100.00%)	18 (90.00%)	
Mixed-echo	0 (0.00%)	2 (10.00%)	
Calcification			0.030*
None	2 (33.30%)	16 (80.00%)	
Yes	4 (66.70%)	4 (20.00%)	
Aspect ratio			0.218
>1	4 (66.70%)	18 (90.00%)	
<1	2 (33.30%)	2 (10.00%)	
Blood			0.197
Absent	1 (16.70%)	10 (50.00%)	
Presence	5 (83.30%)	10 (50.00%)	
2D-SWE			
eEmax			0.001*
0	2 (33.3%)	20 (100.0%)	
1	4 (66.7%)	0 (0.0%)	
eEratio			0.008*
0	3 (50.0%)	20 (100.0%)	
1	3 (50.0%)	0 (0.0%)	
CEUS			
Enhancement mode			0.017*
0	0 (0.0%)	12 (60.0%)	
1	6 (100.0%)	8 (40.0%)	
Perfusion defect			0.028*
0	2 (33.3%)	17 (85.0%)	
1	4 (66.7%)	3 (15.0%)	
Area ratio			0.013*
0	2 (33.3%)	18 (90.0%)	
1	4 (66.7%)	2 (10.0%)	

*P-value indicates significant difference.

## Discussion

Breast cancer is the most common cancer among women. As a routine breast screening for women with dense glands in China, upgrading methods are needed to improve the diagnostic efficacy of US, because the US images of benign and malignant breast neoplasms often overlap, especially in BI-RADS 4 neoplasms, showing a large risk span (2%–95%). In our study, the results showed that the B-mode ultrasonographic features such as internal echo and shape were not enough to distinguish between benign and malignant in BI-RADS 4 breast neoplasms, and margin and calcification showed significant differences. Therefore, the development of examination methods with better diagnostic efficacy is necessary. We found that the addition of neural networks and deep learning was superior in differentiating benign from malignant neoplasms in US images (7, 8). This was an important discovery but will take a lot of work to get it into clinical application. Many researchers have proposed that the nomogram risk-prediction model has satisfying prediction efficiency and great clinical practicability, consistent with our previous research (9). A large number of studies have shown that SWE and CEUS have high clinical value in differentiating benign and malignant breast diseases. However, to our knowledge, no study explored the combination of 2D-SWE, 3D-SWE, and CEUS to research the diagnostic efficacy of benign and malignant breast neoplasms. On this basis, this study focused on the diagnostic efficacy of 2D-SWE, 3D-SWE, CEUS, and their combination in BI-RADS 4 breast neoplasms. The nomogram risk-prediction model was developed using the optimal combination.

**Figure 6:**
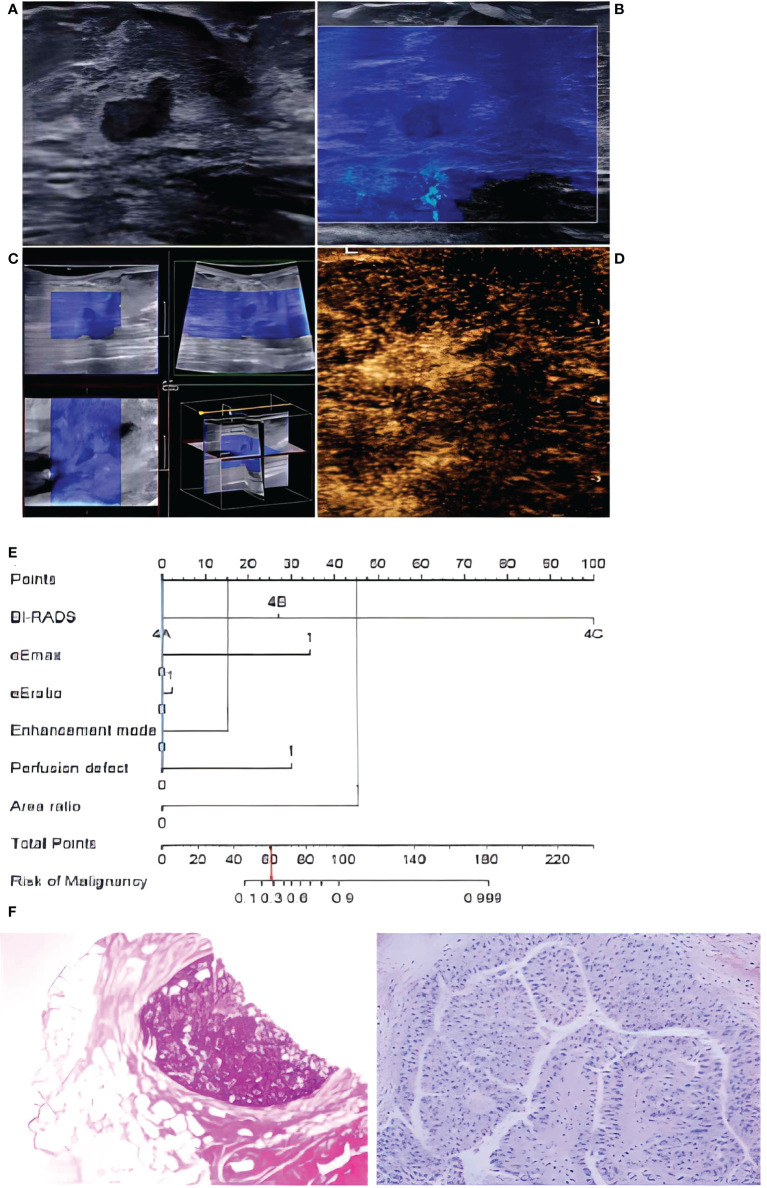
In a 72-year-old woman, lesion size was 0.8 x 0.4 cm in the duct on B-mode imaging **(A)**, considered to be BI-RADS category 4A. The lesion was homogeneously soft (blue color) in 2D-SWE **(B)** and 3D-SWE **(C)**, indicating that it was benign. In CEUS **(D)**, an inhomogeneous hyperenhancement of the lesion was noted, the boundary between the lesion and the surrounding gland was not clear, and the scope was larger than that of the B-mode, indicating malignancy. The risk degree of the nomogram **(E)** was less than 0.3, indicating a benign neoplasm. Pathology **(F)** showed that the lesion was intraductal papilloma.

**Figure 7:**
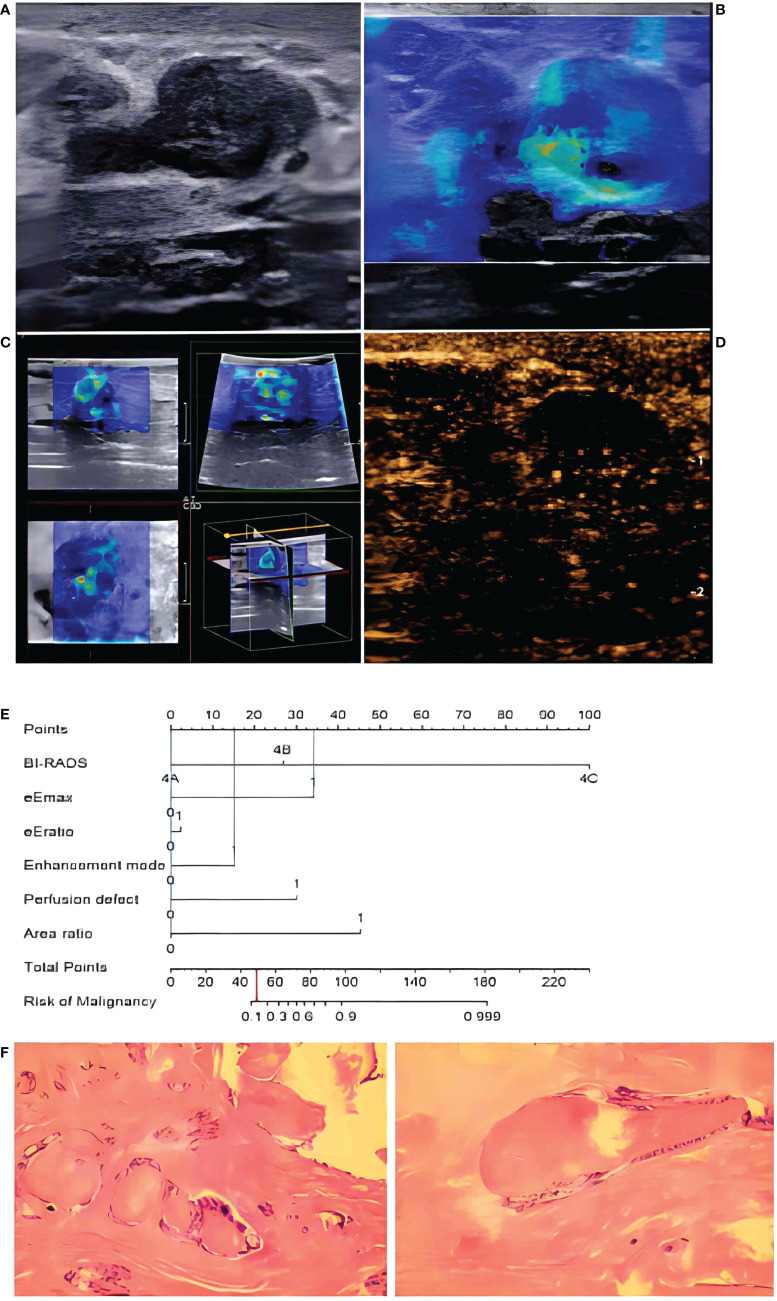
In a 48-year-old woman, B-mode imaging **(A)** showed a 2.0 × 1.1-cm hypoechoic lesion below the nipple, considered to be BI-RADS category 4A. In 2D-SWE **(B)** and 3D-SWE **(C)**, the lesion showed a polychrome or partly hard ring sign, indicating malignancy. In CEUS **(D)**, the lesion showed inhomogeneous low–no enhancement and a well-defined margin, indicating a benign lesion. The risk degree of the nomogram **(E)** was less than 0.2, indicating a benign neoplasm. Pathology **(F)** showed that the lesion was a fibroadenoma.

In 2D-SWE and 3D-SWE, quantitative and qualitative features showed statistical differences, the PPV of quantitative analysis was significantly higher than that of qualitative analysis (93.3, 93.3 vs. 68.2, 75.0), and both of them could significantly improve the accuracy of US. For quantitative analysis, malignant neoplasms had higher values. In 2D-SWE, eEmax (sensitivity: 86.4; specificity: 96.3) and eEratio (sensitivity: 81.8; specificity: 95.1) showed the best performance, consistent with previous studies (5, 10, 11). In color images, the neoplasm showed a hard ring sign significantly indicating malignancy. It might be due to the proliferation of connective tissue or the infiltration of cancer cells into the matrix (12, 13). The consistency of 2D-SWE and 3D-SWE was high. Only 3 of 22 malignant neoplasms did not show this sign (sensitivity: 93.75; specificity: 91.94). The pathological results showed that two cases were ductal carcinoma *in situ*, and one case was invasive lobular carcinoma. The three malignant neoplasms represented an unclear boundary, irregular shape, and high resistance artery blood flow, and microcalcification could be seen on US images in two cases, indicating the importance of US. When there were no signs of malignancy in SWE, US should be given priority. Some researchers believed that the softness of some malignant neoplasms was responsible for the false-negative results, such as ductal carcinoma *in situ*, mucinous carcinoma, etc. (14–16). It should be noted that malignant neoplasms with false-negative results in SWE showed inhomogeneous enhancement in CEUS, with perfusion defect in two cases and enlarged area in two cases. When US showed malignant signs, increasing SWE and CEUS could improve their detection ability of malignant neoplasms and their diagnostic efficacy (**Figures 6**, **7**). In 2D-SWE and 3D-SWE, five and seven cases of benign neoplasms were false-positive, respectively. The pathological results showed that all the neoplasms were fibroadenoma or adenosis with a diameter above 1.5 cm, and the false-positive rate of 3D-SWE was higher. According to Youk et al., this might be related to the convex shape and weight of the 3D probe (17). Barr et al. and Balleyguier et al. noted that fibroadenoma was common in SWE false-positive results, which might be because hardened and proliferated tissues increased shear-wave velocity (14, 15). The size of neoplasms was also an important factor in SWE false-positive results (18, 19). We considered that SWE could improve the diagnostic accuracy of US and the diagnostic confidence of radiologists and also contribute to patient management by clinicians, which was approved by the BE1 prospective study in multiple nations (20).

The results showed that US+2D-SWE, US+3D-SWE, and US+2D-SWE+3D-SWE could significantly improve the diagnostic efficacy of US in the diagnosis of BI-RADS 4 breast neoplasms, but the difference was not statistically significant. The diagnostic efficiency of US+3D-SWE was not better than that of US+2D-SWE, and US+2D-SWE+3D-SWE has no significant improvement in the diagnostic efficiency of US+2D-SWE and US+3D-SWE. 2D-SWE takes 2–3 min, while 3D-SWE takes 10–15 min, which is longer, so we think that 2D-SWE is more adaptable in clinical practice. Lee et al. studied the application of US, 2D-SWE, and 3D-SWE in breast neoplasms (21). They think that both 2D-SWE and 3D-SWE showed similar diagnostic performance, and both of them could significantly increase the AUC of US. The performance of US+3D-SWE was not better than that of US+2D-SWE, which was consistent with our research results. Youk et al. thought that 2D-SWE performed better (17), probably because we included Eratio in the quantitative analysis. There was no difference in diagnostic efficacy between US+2D-SWE and US+3D-SWE. Tian et al. believed that the quantitative characteristics of 3D-SWE and 2D-SWE could significantly improve the diagnostic performance of US, especially the Esd of 3D-SWE which had considerable clinical value (22), which was not consistent with our point of view, and this may be because they only studied Esd and Emax. Although 3D-SWE is not superior to 2D-SWE, we consider it to be of help when radiologists lack diagnostic confidence.

The value of CEUS in the diagnosis and evaluation of the curative effect of breast cancer has been recognized (23, 24). Our research revealed that malignant neoplasms usually showed high heterogeneous enhancement, unclear enhancement boundary, perfusion defect, perforating vessels, large post-enhanced area, and high PI and tAUC values. In contrast, most benign breast neoplasms often presented equal or low homogeneous enhancement on CEUS, and the boundary was clear after enhancement. Among them, enhancement mode, perfusion defect, and area ratio represented the optimum prediction accuracy, which was relevant to the unique growth characteristics of tumors. Malignant tumors usually proliferate rapidly with more abundant microvessels and tend to invade outwards (25, 26), often showing higher enhancement and larger area than US images. Insufficient blood supply often causes tumor anoxia and necrosis, and while CEUS is pure blood pool imaging, necrotic tissues without blood flow show perfusion defects, making the imaging different from benign neoplasms. This cannot be realized by US. Some benign neoplasms (intraductal papilloma, fibroadenoma, and inflammatory neoplasms) also presented as malignant neoplasms in our study, with high heterogeneous enhancement, perfusion defect, and larger area. This might be attributed to the increase and proliferation of cells in benign neoplasms. Especially when there is inflammatory cell infiltration, it is challenging to distinguish inflammatory neoplasms from malignant neoplasms because of their biological characteristics (27). Some researchers believe that CEUS is less effective in identifying fibroadenoma, ductal papilloma, and low-grade intraductal carcinoma, especially neoplasms with a size smaller than 1 cm (28). There was no statistical difference between CEUS qualitative analysis and quantitative analysis. In contrast, some researchers insist that the diagnostic effectiveness of qualitative analysis is better, probably because they do not include the area ratio in the quantitative analysis or consider the diameter in the qualitative analysis (29, 30). The enlargement of the malignant neoplasm area in CEUS has a high diagnostic value (31). CEUS can help both junior and senior radiologists to improve their diagnostic accuracy (32), and US+CEUS is more effective than US in the differential diagnosis of breast neoplasms (33).

US+2D-SWE+CEUS could significantly improve the diagnostic efficacy of US+2D-SWE and US+CEUS, and there was no significant difference compared with US+3D-SWE+CEUS and US+2D-SWE+3D-SWE+CEUS. 3D-SWE was time-consuming and showed a higher requirement for operators, making it more easily to cause false-positive results due to artificial rigidity. Considering clinical practicability, US+2D-SWE+CEUS was more applicable. The study of Liu et al. believed that both US+2D-SWE+CEUS and US+2D-SWE could significantly improve the accuracy of US in diagnosing BI-RADS 4 breast neoplasms, but there was no significant difference in AUC (6). This may be due to the inclusion of CEUS quantitative analysis (TIC) in our study. In addition, we added 3D-SWE to explore the optimal combination with great diagnostic performance for BI-RADS 4 breast neoplasms.

The nomogram based on US+2D-SWE+CEUS performs well in the training cohort and the validation cohort and has an AUC of over 0.94 for both. US+2D-SWE+CEUS shows high diagnostic efficiency, but 17 ultrasound features and the BI-RADS category need to be considered, while the nomogram only needs to consider 5 ultrasound features and the BI-RADS category, which ensures a high AUC value and reduces the need for considering the features of the ultrasound; thus, it can help radiologists to do their work efficiently and accurately.

This study had some limitations, including a small sample size, limited breast disease scope, and being a single-center study. On this basis, it is necessary to expand the sample size and conduct a cross-group comparison of a multicenter prospective study. Additionally, there was no confounding factor analysis in this study, and age, the maximum diameter of the neoplasm, and pathological type were not taken into account. Some widely accepted factors affecting diagnostic performance, such as histological differentiation, breast type, and gland thickness, were not evaluated. In addition, patients without pathological examination were excluded, which might lead to selection bias. Since this study was performed in an authentic clinical environment, interobserver and intraobserver consistency were not fully assessed. It is recognized that SWE and CEUS are highly repeatable (34, 35). Before the SWE examination, the radiologist accepted training on ways to reduce technical errors, such as manual compression and transducer movement, and a CEUS examination was performed by an experienced radiologist. According to the present study design, the images were reviewed by two radiologists, and cases of poor image quality were screened out before analysis. Therefore, we assumed that interobserver and intraobserver variation might have no significant effect on our results.

## Conclusion

US, 2D-SWE, 3D-SWE, CEUS, and their combination could improve the diagnostic efficiency of BI-RADS 4 breast neoplasms. US+2D-SWE+CEUS showed the optimal diagnostic performance. The nomogram based on US+2D-SWE+CEUS performs well and can help clinicians manage BI-RADS 4 breast neoplasms. Although the diagnostic efficacy of US+3D-SWE is not superior to that of US+2D-SWE, some characteristic features are helpful in the diagnosis of malignant breast nodules.

## Data availability statement

The raw data supporting the conclusions of this article will be made available by the authors, without undue reservation.

## Ethics statement

The studies involving human participants were reviewed and approved by the Ethics Committee of Xiangya Hospital Central South University (No.202010143) and the Chinese Clinical Trial Registry (ChiCTR2100050604). The patients/participants provided their written informed consent to participate in this study.

## Author contributions

Guarantors of the integrity of the entire study: YC, JRL, JL, XH, JTL, and BZ. Literature research: YC, JRL, and BZ. Study concepts/study design: BZ. Contributed to the acquisition of data: all authors. Clinical studies: YC, JRL, JL, XH, and BZ. Contributed reagents/materials/analysis tools: YC, JTL, and BZ. Manuscript drafting or manuscript revision: YC and BZ. Statistical analysis: YC, JRL, JL, XH, and BZ. All authors contributed to the article and approved the submitted version.

## Conflict of interest

The authors declare that the research was conducted in the absence of any commercial or financial relationships that could be construed as a potential conflict of interest.

## Publisher’s note

All claims expressed in this article are solely those of the authors and do not necessarily represent those of their affiliated organizations, or those of the publisher, the editors and the reviewers. Any product that may be evaluated in this article, or claim that may be made by its manufacturer, is not guaranteed or endorsed by the publisher.
